# ﻿Three new species of *Dicephalospora* (Leotiomycetes, Helotiales) from Northern Thailand and Southwestern China

**DOI:** 10.3897/mycokeys.115.143994

**Published:** 2025-03-11

**Authors:** Le Luo, Kandawatte Wedaralalage Thilini Chethana, Qi Zhao, Vinodhini Thiyagaraja, Kitiphong Khongphinitbunjong, Fatimah Al-Otibi, Kevin D. Hyde

**Affiliations:** 1 State Key Laboratory of Phytochemistry and Natural Medicines, Kunming Institute of Botany, Chinese Academy of Sciences, Kunming 650201, Yunnan, China; 2 Center of Excellence in Fungal Research, Mae Fah Luang University, Chiang Rai 57100, Thailand; 3 School of Science, Mae Fah Luang University, Chiang Rai 57100, Thailand; 4 Guiyang Institute of Humanities and Technology, Guiyang 550025, China; 5 Department of Botany and Microbiology, College of Science, King Saud University, P.O. Box 22452, Riyadh 11495, Saudi Arabia

**Keywords:** 3 new species, Helotiaceae, morphology, phylogeny, taxonomy

## Abstract

*Dicephalospora* is a discomycetous genus belonging to the family Helotiaceae (Helotiales). The genus currently comprises 19 species. Among them, 17 species have been reported from Asia, of which 14 were reported from China. During a survey of Leotiomycetes, we collected six specimens of *Dicephalospora* species from southwest China and northern Thailand. The samples were examined based on the phylogenetic analyses of LSU and ITS sequence data, along with morphological characterization, and our results demarcate three new *Dicephalospora* species, viz., *D.tengyueica*, *D.maetaengica* and *D.menghaica*. *Dicephalosporatengyueica***sp. nov.** formed a distinct clade closely related to *D.rufocornea*, *D.irregularis* and *D.sagerae*, whereas *D.maetaengica***sp. nov.** clustered closer to *D.menghaica***sp. nov.** with 100% ML and 1.00 BIPP statistical support. All three species have similar apothecia sizes but differ in their coloration. *Dicephalosporatengyueica* has aseptate ascospores, while *D.menghaica* and *D.maetaengica* have 7-septate ascospores. The paraphyses in *D.menghaica* are branched, while they are unbranched in the other two species. An updated key to the known species of *Dicephalospora* is also provided.

## ﻿Introduction

*Dicephalospora* belongs to Helotiaceae (Helotiales) and was described by Spooner in 1987, with *D.calochroa* (Syd. and P. Syd.) Spooner as the type species (Spooner 1987; Hyde et al. 2024). The taxonomy of the Helotiales has undergone multiple revisions, driven largely by advances in molecular phylogenetics (Ekanayaka et al. 2019; Johnston et al. 2019). Two gene regions, ITS and LSU, have been primarily used in the phylogenetic analyses of Helotiales, further supporting the inclusion of *Dicephalospora* within Helotiaceae (Han et al. 2014; Zhao et al. 2016; Ekanayaka et al. 2019). Helotiaceae is the most heterogeneous family in Helotiales and is characterized by parallel hyphae, angular to isodiametric cells, interwoven hyphae, and margins and flanks that may be covered with hairs or completely absent (Groves 1968; Korf 1973; Wijayawardene et al. 2022). The taxonomy of Helotiales, including Helotiaceae, has been widely researched and debated. Notably, a multi-gene phylogenetic analysis incorporating up to 15 genes across 279 specimens has provided a more resolved, refined classification framework for Leotiomycetes, including members of Helotiaceae (Johnston et al. 2019). After multiple revisions, a total of 26 genera are now accepted within the family, many of which are saprobic on plant material (Ekanayaka et al. 2019; Wijayawardene et al. 2022).

Morphologically, *Dicephalospora* species are distinct due to their erumpent or superficial, stipitate apothecia, which can vary in color from yellow and orange to red and blackish. The ectal excipulum comprises "*textura prismatica*" cells with refractive walls, while the medullary excipulum consists of cells of "*textura intricata*". The asci are filiform and show variable reactions in Melzer’s reagent (J+ or J-). The paraphyses are straight or slightly curved at the apex, and the ascospores are hyaline, sub-ellipsoid to fusoid, guttulate, and often have a mucilaginous cap at the poles (Hosoya et al. 1999; Zhuang et al. 2016). The taxa are predominantly distributed in Asia (Phutthacharoen et al. 2022), with 17 of 19 listed species reported from this region (Luo et al. 2024). Exceptionally, *D.chrysotricha* is reported from New Zealand (Zheng and Zhuang 2019), *D.sagerae* is reported from Australia, and *D.calochroa* shows extended distribution and is reported in China and Papua New Guinea (Spooner 1987). So far, 14 species (viz., *D.albolutea*, *D.aurantiaca*, *D.calochroa*, *D.contracta*, *D.damingshanica*, *D.dentata*, *D.huangshanica*, *D.phaeoparaphysis*, *D.pinglongshanica*, *D.rufocornea*, *D.sessilis*, *D.shennongjiana*, *D.xishuangbannaensis* and *D.yunnanica*) have been reported from China (Phutthacharoen et al. 2022; Luo et al. 2024).

Hosoya et al. (1999) discovered dicephalosterol from the culture of *D.rufocornea*, which has a potential application for prostatic hypertrophy. However, the applications of *Dicephalospora* species are poorly explored due to the difficulty in obtaining pure cultures and their slow growth on artificial media, as well as their minimal biomass in nature (Zheng and Zhuang 2019).

*Dicephalospora* represents a morphologically distinct and phylogenetically significant genus within Helotiaceae. While challenges remain in cultivating these fungi and exploring their biochemical potential, ongoing molecular studies continue to advance our understanding of their taxonomy, ecology, and potential applications. During the investigation of Leotiomycetes, six collections of *Dicephalospora* were obtained from southwest China and northern Thailand. Morphological and phylogenetic analyses based on LSU and ITS data were performed to confirm their taxonomic placement, which revealed three new *Dicephalospora* species based on a polyphasic approach, along with an updated dichotomous key for the genus.

## ﻿Material and methods

### ﻿Collection and morphological examinations

Six specimens were collected from southwest China and northern Thailand, primarily in highly humid, natural broadleaf forests and protected areas with minimal human interference. The fruiting bodies were found on the surface of decaying wood and were photographed in the field before placing them in plastic containers for transport to the laboratory. The samples were dehydrated using a dehydrator set to a temperature between 25–30 °C. Further, the samples were examined using morphological and phylogenetic analyses. For detailed morphological examination, dried specimens were observed under a stereomicroscope (C-PSN, Nikon, Japan) with images captured using a digital camera (Canon EOS 70D, Japan) attached to the microscope. Free-hand sections of the dried specimens were mounted in water to observe microscopic characteristics such as apothecia, exciple, paraphyses, asci, and ascospores using a Nikon compound microscope (Nikon, Japan) with a DS-Ri2 camera. Sections were also treated with Melzer’s reagent for the iodine test (Tochihara and Hosoya 2022). Measurements of the microstructures were taken using the Tarosoft (R) Image Frame Work program v.0.97 (Tarosoft, Thailand). These measurements are presented in the format (a–) b–c(–d), where ‘a’ represents the minimum value, ‘d’ the maximum value, and ‘b–c’ the 90% confidence interval. The specimens were deposited in the Cryptogamic Herbarium of the Kunming Institute of Botany, Chinese Academy of Sciences (KUN-HKAS), and the Mae Fah Luang University Herbarium (Herb. MFLU). The Facesoffungi and the Index Fungorum numbers were obtained following the procedures outlined by Jayasiri et al. (2015) and Index Fungorum (2025), respectively. The morphological description and the phylogenetic tree of the new species were submitted to the Greater Mekong Subregion webpage (Chaiwan et al. 2021). Images used for figures were processed with Adobe Photoshop CS6 Extended version 13.0 × 64 (Adobe Systems, USA).

### ﻿DNA extraction, PCR amplifications and sequencing

Genomic DNA was extracted from the dried apothecia using a TSP101 DNA extraction kit (TSINGKE, China). Following the latest studies (Phutthacharoen et al. 2022; Luo et al. 2024), LSU and ITS regions were subjected to PCR amplification, using the primers LR0R/LR5 (Vilgalys and Hester 1990) and ITS1/ITS4 (White et al. 1990; Gardes and Bruns 1993), respectively. The total volume of PCR amplifications was 25 μL, including 12.5 μL of 2X PCR G013 Taq MasterMix with Dye (Applied Biological Materials, Canada), 1 μL of each primer (10 μM), 2 μL of genomic DNA, and 8.5 μL of sterilized, distilled water. Amplifications were conducted under the following conditions: pre-denaturation at 95 °C for 5 minutes, followed by 35 cycles of denaturation at 95 °C for 20 seconds, annealing at 55 °C for 10 seconds, elongation at 72 °C for 20 seconds and final elongation at 72 °C for 7 minutes. Gel electrophoresis with 1% TAE and TSJ003 GoldView nucleic acid dye (TSINGKE, China) was used to test the obtained PCR products. Finally, the PCR products were sequenced by Tsingke Biotechnology Co., Ltd., Kunming, China. Newly produced sequences were deposited in the GenBank and the accession numbers were given in Table 1.

**Table 1.** The taxa included in the phylogenetic analysis along with their corresponding GenBank accession numbers. Newly identified taxa are in bold. Names with (^T^) indicate type specimens and ‘-’ denotes unavailable data in the GenBank.

Species	Strain	GenBank Accession No.		Reference
		ITS	LSU	
*Amylocarpusencephaloides*	CBS 129.60	MH857920	MH869464	Vu et al. (2019)
*Amylocarpusencephaloides*	017cN	KM272369	KM272361	Rämä et al. (2014)
*Bryoscyphusdicrani*	M141	EU940183	EU940107	Stenroos et al. (2010)
*Connersiarilstonii*	CBS 537.74	KJ755499	AF096189	Suh and Blackwell (1999)
*Crocicreasamenti*	F-147481	FJ005093	FJ005124	Peláez et al. (2011)
*Crocicreascacaliae*	F-148706	FJ005107	FJ005126	Peláez et al. (2011)
*Crocicreascyathoideum*	MFLU 18-0698	MK584943	MK591970	Ekanayaka et al. (2019)
*Crocicreastomentosum*	MFLU 17-0082	MK584988	MK592008	Ekanayaka et al. (2019)
*Cudoniellaclavus*	AFTOL-ID 166	DQ491502	DQ470944	Spatafora et al. (2006)
*Cyathiculamicrospora*	M267	EU940165	EU940088	Baral et al. (2009)
*Dicephalosporaalbolutea*	HMAS 279693	MK425601	–	Zheng and Zhuang (2019)
*Dicephalosporaaurantiaca*	MFLU 16-0591a	MK584962	MK591988	Ekanayaka et al. (2019)
*Dicephalosporachiangraiensis*	MFLU 21-0019	MZ241818	MZ241827	Phutthacharoen et al. (2022)
*Dicephalosporachiangraiensis*	MFLU 21-0018^(T)^	MZ241817	MZ241826	Phutthacharoen et al. (2022)
*Dicephalosporachrysotricha*	PDD:91762	KF727411	–	Unpublished
*Dicephalosporachrysotricha*	PDD:58197	KF727409	–	Unpublished
*Dicephalosporachrysotricha*	PDD:93932	MH578487	–	Unpublished
*Dicephalosporachrysotricha*	PDD:81537	KF727410	OQ466391	Unpublished
*Dicephalosporadentata*	3093	KP204263	–	Liu et al. (2016)
*Dicephalosporahuangshanica*	MFLU 18-1828	MK584979	MK591979	Ekanayaka et al. (2019)
*Dicephalosporahuangshanica*	KUS-F52405	JN033408	JN086711	Han et al. (2014)
*Dicephalosporainthanonensis*	MFLU 22-0050^(T)^	ON606312	ON604634	Phutthacharoen et al. (2022)
*Dicephalosporainthanonensis*	MFLU 22-0053	ON606313	ON604635	Phutthacharoen et al. (2022)
*Dicephalosporairregularis*	MFLU 22-0054^(T)^	ON511117	ON514038	Phutthacharoen et al. (2022)
***Dicephalosporamaetaengica***	**MFLU24-0330** ^(T)^	**PQ481904**	**PQ481910**	This study
***Dicephalosporamaetaengica***	**MFLU24-0331**	**PQ481905**	**PQ481911**	This study
***Dicephalosporamenghaica***	**HMAS 135692** ^(T)^	**PQ481908**	**PQ481914**	This study
***Dicephalosporamenghaica***	**HMAS 135690**	**PQ481909**	**PQ481915**	This study
*Dicephalosporarufocornea*	MFLU 16-1860	MK584989	MK592011	Ekanayaka et al. (2019)
*Dicephalosporarufocornea*	MFLU 19-2083	MZ241816	MZ241825	Phutthacharoen et al. (2022)
*Dicephalosporarufocornea*	TNS:F:36242	LC136911	–	Unpublished
*Dicephalosporarufocornea*	TNS:F:40155	LC136918	–	Unpublished
*Dicephalosporarufocornea*	MFLU 16-1858	MK584991	–	Ekanayaka et al. (2019)
*Dicephalosporarufocornea*	FCATAS5710	PP622049	–	Unpublished
*Dicephalosporasagerae*	BRIP 72428d	NR_182617	–	Tan and Shivas (2022)
*Dicephalosporasessilis*	MFLU 18-1823^(T)^	NR_163779	NG_068621	Ekanayaka et al. (2019)
*Dicephalosporashennongjiana*	HMAS 279698	MK425606	–	Zheng and Zhuang (2019)
*Dicephalosporaxishuangbannaensis*	HMAS 131157	OR948047	–	Luo et al. (2024)
*Dicephalosporaxishuangbannaensis*	HMAS 131164^(T)^	OR948048	–	Luo et al. (2024)
***Dicephalospora* tengyueica**	**HMAS 135691** ^(T)^	**PQ481906**	**PQ481912**	This study
***Dicephalospora* tengyueica**	**HMAS 135694**	**PQ481907**	**PQ481913**	This study
*Dicephalosporayunnanica*	HMAS 279701	MK425609	–	Zheng and Zhuang (2019)
*Dicephalosporayunnanica*	HMAS 279700	MK425608	–	Zheng and Zhuang (2019)
*Dicephalosporayunnanica*	HMAS 61850	DQ986486	–	Zheng and Zhuang (2019)
*Endoscyphaperforans*	PDD:102231	KF727424	MK039717	Unpublished
*Glarealozoyensis*	ATCC 20868^(T)^	NR_137138	–	Bills et al. (1999)
*Glarea* sp.	C2B	KX610435	–	Yokoya et al. (2017)
*Gloeotiniagranigena*	CBS 417.50	–	MH868212	Vu et al. (2019)
*Hymenoscyphusfructigenus*	CBS 186.47	MH856211	MH867741	Vu et al. (2019)
*Hymenoscyphusoccultus*	KUS-F52847	KP068064	–	Gross and Han (2015)
*Hymenoscyphuspseudoalbidus*	Hokk_14	KJ511191	–	Gross et al. (2014)
*Hymenotorrendiellaeucalypti*	PDD:70105	MH578483	–	Unpublished
*Hymenotorrendiellaeucalypti*	CPC 11050^(T)^	DQ195788	DQ195800	Zhao (2014)
*Lanziaberggrenii*	ICMP:19614	KC164645	KC164640	Johnston and Park (2013)
*Ombrophilaviolacea*	WZ0024	AY789366	AY789365	Wang et al. (2005)
*Phaeohelotiumepiphyllum*	TNS: F-40042	AB926061	AB926130	Zhao (2014)
*Pirottaeapalmicola*	PDD:60282	KM677208	–	Unpublished
*Pirottaeapalmicola*	PDD:65971	KM677206	–	Unpublished
*Pleuroascusnicholsonii*	CBS 345.73	KJ755519	AF096196	Zhao (2014)
*Roesleriasubterranea*	CBS 339.96	EF060308	EF608074	Kirchmair et al. (2008)
*Roesleriasubterranea*	CBS 407.51	MH856922	–	Vu et al. (2019)

### ﻿Phylogenetic analyses

Newly generated DNA sequences were assembled using BioEdit v.7.2.5 (Hall 1999) to obtain consensus sequences. The concatenated sequences were used to search for the close relatives in the NCBI (Johnson et al. 2008). The closely related sequences were downloaded from GenBank following the recent papers (Phutthacharoen et al. 2022; Luo et al. 2024) (Table 1). *Pleuroascusnicholsonii* (CBS 345.73) and *Connersiarilstonii* (CBS 537.74) were selected as outgroup taxa. The phylogenetic analysis was conducted based on the datasets, including reference DNA sequences and newly generated DNA sequences using OFPT (Zeng et al. 2023) with the following protocol. Datasets of each gene region were first independently aligned with the ‘auto’ strategy (based on data size) by MAFFT (Katoh and Standley 2013) and trimmed with the ‘gappyout’ option (based on gaps’ distribution) by TrimAl (Capella-Gutiérrez et al. 2009). The best-fit nucleotide substitution models for each dataset were then selected based on the Bayesian information criterion (BIC) from twenty-two common DNA substitution models with rate heterogeneity by ModelFinder (Kalyaanamoorthy et al. 2017). All datasets were concatenated with partition information for the subsequent phylogenetic analyses. Maximum likelihood with 1000 replicates was performed using ultrafast bootstrap approximation (Hoang et al. 2018) with SH-like approximate likelihood ratio test (SH-aLRT) (Guindon et al. 2010) by IQ-TREE (Nguyen et al. 2015). The consensus tree was summarized based on the extended majority rule. Bayesian inference was performed with two parallel Metropolis-coupled (one ‘cold’ chain and three heated chains) Markov chain Monte Carlo runs by MrBayes (Ronquist et al. 2012), with trees sampling every 100^th^ generations. The consensus tree was summarized after discarding the first 25% of samples when the average standard deviation of split frequencies fell below 0.01. The resulting trees were visualized in FigTree v1.4.3 (http://tree.bio.ed.ac.uk/software/figtree/). The polyphasic approach recommended by Chethana et al. (2021) was followed to establish the new species.

## ﻿Results

### ﻿Phylogenetic analysis

**Figure 1.**
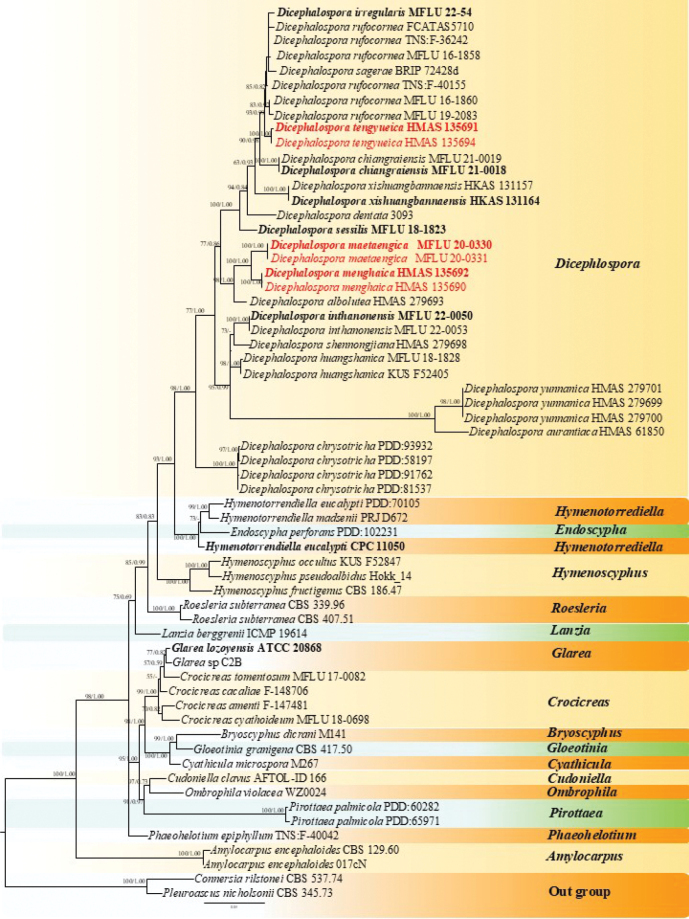
The IQ-Tree phylogram based on the combined LSU and ITS dataset. The MLBS ≥ 70% and BIPP ≥ 0.90 are shown at the nodes as MLBS/BIPP. The MLBS < 70% and BPP < 0.90 are expressed as a hyphen (“-”). Type strains are in bold. Names in red indicate isolates from the current study.

The data matrix consisted of LSU and ITS sequences, representing 62 taxa distributed across 17 genera of Helotiaceae with 1192 characters after trimming, including gaps (ITS: 404 bp and LSU: 788 bp). The matrix had 390 distinct alignment patterns, with 28.73% undetermined characters or gaps. Estimated parameters for the tree are as follows: total tree length (sum of branch lengths): 1.4788; sum of internal branch lengths: 0.9205 (62.2453% of tree length); gamma distribution shape parameter *α* = 0.7196. The best IQ-Tree with a final likelihood value of -7128.2117 is presented in Fig. 1. The tree topology inferred from ML analysis is similar to that of BI analysis. Index Fungorum (2025) listed 19 species epithets for *Dicephalospora*, and 15 of them are included in our phylogenetic analyses (Fig. 1), and four species (*D.calochroa*, *D.damingshanica*, *D.pinglongshanica* and *D.phaeoparaphysis*) are excluded due to a lack of molecular data. The phylogenetic result showed that *Dicephalospora* species clustered together and concurred with the previous studies (Zheng and Zhuang 2019; Phutthacharoen et al. 2022; Luo et al. 2024). *Dicephalospora* species formed a monophyletic clade separated from an assemblage of *Endoscypha* and *Hymenotorrendiella* taxa by 93% maximum likelihood bootstrap support (MLBS) and 1.00 Bayesian inference posterior probability (BIPP) support. The new species *D.tengyueica* formed a distinct clade closely related to *D.rufocornea*, *D.irregularis*, and *D.sagerae*, with 93% MLBS and 0.99 BIPP support (Fig. 1). The two new species, *D.maetaengica* and *D.menghaica*, were closely related, supported by 100% MLBS and 1.00 BIPP, and together formed a sister clade to *D.albolutea*, with 98% MLBS and 1.00 BIPP support (Fig. 1).

### ﻿Taxonomy

#### ﻿ Dicephalospora tengyueica

Taxon classificationFungiHelotialesHelotiaceae

L. Luo & K.D. Hyde sp. nov.

E8E0A4B0-2659-5C75-AACD-1E2B45F4607F

Index Fungorum: IF902889

Facesoffungi Number: FoF16773

Fig. 2

##### Etymology.

The epithet “*tengyueica*” refers to the collection site, Tengyue street, where the holotype specimen was collected.

##### Holotype.

HKAS135691.

##### Description.

***Saprobic*** on dead twigs. **Sexual morph**: ***Apothecia*** 1.2–3.2 mm diam., when dry arising solitary or gregarious in a small group, scattered on wood, centrally stipitate, superficial, orange when fresh, become light brown when dry. ***Stipe*** 0.9–2.3 mm height at the base, yellow when fresh, and light yellow to white when dry. ***Receptacle*** orange and discoid. ***Margins*** slightly rough, orange to dark orange. ***Disc*** slightly convex and orange. ***Ectal excipulum*** 30–66 µm (x̄ = 46 µm, n = 20), multi-layered, and thin-walled, with hyaline cells of ***textura globulosa***. ***Medullary excipulum*** 21–48 µm (x̄ = 33 µm, n = 30), composed of thin-walled, hyaline, gelatinized cells of ***textura globulosa*** to ***porrecta***, small cells condensed. ***Hymenium*** 84–171 µm (x̄ = 131 µm, n = 60), hyaline to yellowish, inner mixed with asci and paraphyses. ***Paraphyses*** 1.1–3.2 µm wide (x̄ = 1.9 µm, n = 30), at the terminal cell, filiform, numerous, lengths exceeding the asci, unbranched, aseptate, apical cells swollen and globose, filled with oil droplets. ***Asci*** (85–)90–118(–125) × 5.9–12.5 µm (x̄ = 102 × 9.5 µm, n = 50), eight-spored, unitunicate, cylindrical, and clavate, J- in Melzer’s reagent, tapered long stipitate base. ***Ascospores*** (17–)19–32(–35) × 2–3.5 µm (x̄ = 27.4 × 2.9 µm, n = 60), uniseriate to biseriate, fusiform, aseptate, both ends are filled with oil droplets of different sizes, some narrowed ends capped with a small and gelatinous collar. **Asexual morph**: Not observed.

**Figure 2.**
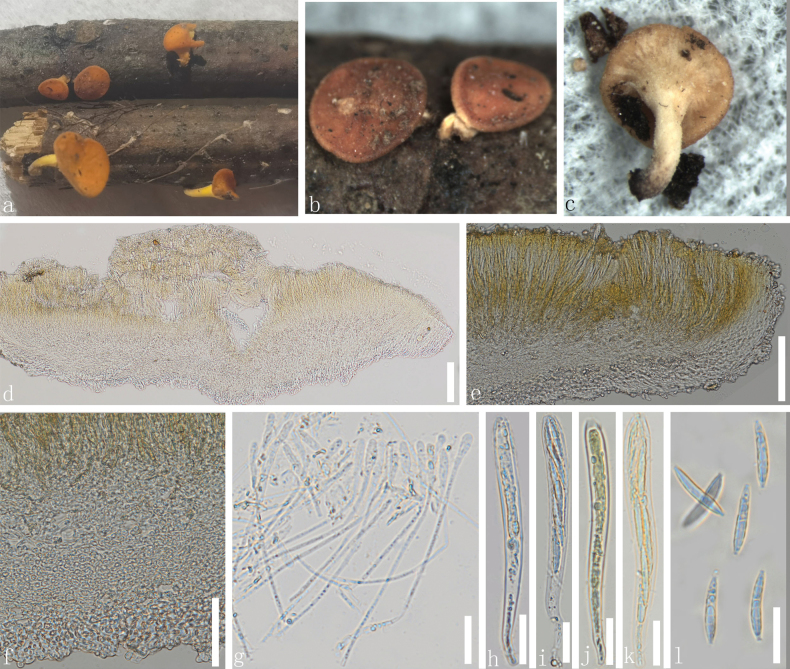
*Dicephalosporatengyueica* (HKAS 135691, holotype) **a** fresh apothecia growing on dead wood **b**, **c** dried apothecia **d**, **e** vertical sections of an apothecia **f** excipulum **g** paraphyses **h–k** asci (**j–k** asci in Meltzer’s reagent) **l** ascospores. Scale bars: 5 mm (**a**); 2 mm (**b, c**); 100 µm (**d, e**); 50 µm (**f**); 20 µm (**g–l**).

##### Material examined.

China • Yunnan Province, Tengchong City, Tengyue Street, altitude 1749 m, on the decayed unidentified twigs, 19 August 2022, Le Luo, ly289 (HKAS 135691, holotype); • *ibid*., ly290 (HKAS 135694, paratype).

##### Notes.

Our specimens of *D.tengyueica* (HKAS 135691 and HKAS 135694) formed a distinct clade (93% MLBS and 0.99 BIPP), closely related to *D.rufocornea*, *D.irregularis* and *D.sagerae* (Fig. 1). However, the new species exhibit distinct morphological features in having orange to light brown apothecia, light yellow stipe, tapered long stipitate base, aseptate ascospores, and aseptate paraphyses. *Dicephalosporarufocornea* has red or reddish-orange apothecia, red stipe, and sessile base with septate paraphyses (Ekanayaka et al. 2019), whereas *D.irregularis* has sessile apothecia, asci arising from simple septa without basal protuberance, aseptate to septate ascospores that are wider than *D.tengyueica* (5.5–7.5 µm vs. 2–3.5 µm) (Phutthacharoen et al. 2022). The detailed morphological description is not available for *D.sagerae* (Tan & Shivas, 2022) but it differs from *D.tengyueica* in the ITS base pair comparison, which revealed 8.5% differences. Therefore, *D.tengyueica* is introduced here as a new species.

#### ﻿ Dicephalospora maetaengica

Taxon classificationFungiHelotialesHelotiaceae

L. Luo & K.D. Hyde sp. nov.

CA87B806-2823-58B8-8DCA-FFC6525A94C8

Index Fungorum: IF902890

Facesoffungi Number: FoF16774

Fig. 3

##### Etymology.

The epithet “*maetaengica*” refers to the collection site, MaeTaeng District, where the holotype specimen was collected.

##### Holotype.

MFLU 24-0330.

##### Description.

***Saprobic*** on dead leaves. **Sexual morph**: ***Apothecia*** 1.5–3.3 mm diam., when dry arising solitary, uniseriate on the stem of the leaves, centrally long stipitate, superficial, yellow when fresh and dry. ***Stipe*** 1.2–2.5 mm height, slightly hyaline to light yellow at the base. ***Receptacle*** yellow and cupulate. ***Margins*** smooth, yellow to pale yellow. ***Disc*** slightly sunken and yellow. ***Ectal excipulum*** 23–44 µm (x̄ = 33 µm, n = 60), multi-layered, thin-walled, hyaline to light yellow or pale green cells of ***textura globulosa***, slightly larger cells condensed. ***Medullary excipulum*** 28–61 µm (x̄ = 45 µm, n = 60), composed of thin-walled, hyaline to yellowish, gelatinized cells of ***textura porrecta*** to ***globulosa***, small cells condensed. ***Hymenium*** 99–146 µm (x̄ = 123 µm, n = 60), hyaline to yellow, inner mixed with asci and paraphyses. ***Paraphyses*** 1.1–2.1 µm wide (x̄ = 1.6 µm, n = 60), filiform, numerous, lengths exceeding the asci, unbranched, aseptate, slightly swollen, small, globose at the apex. ***Asci*** (75–)81–111(–118) × 5.6–10.7 µm (x̄ = 99 × 8.5 µm, n = 50), eight-spored, unitunicate, cylindrical, clavate, amyloid (J+) having a rounded apex in Melzer’s reagent, tapered long stipitate base. ***Ascospores*** (12.5–)14.5–21.8(–22.5) × 2.4–4.2 µm (x̄ = 18.2 × 3.3 µm, n = 60), uniseriate to biseriate, fusiform, guttulate, 0–1–septate. **Asexual morph**: Not observed.

**Figure 3.**
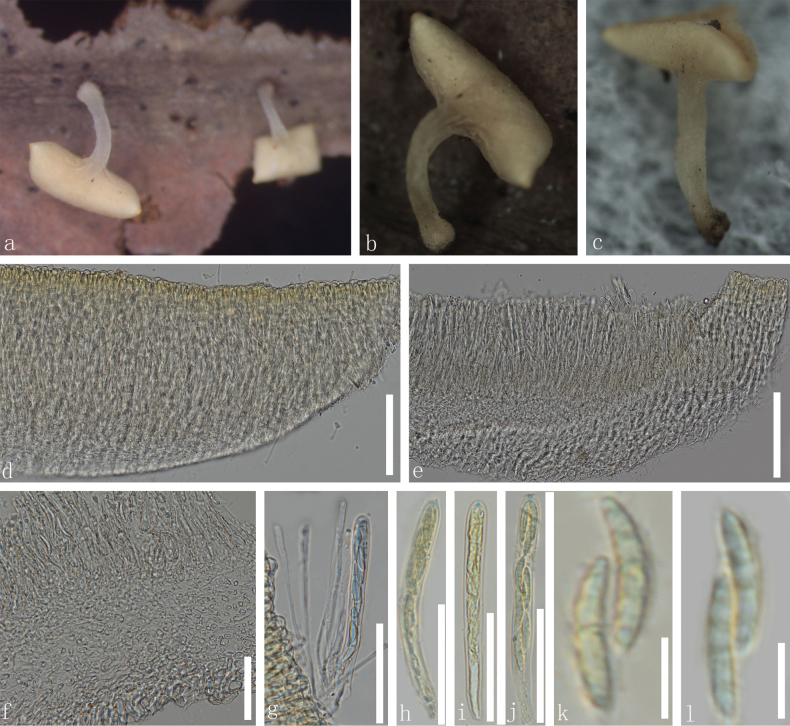
*Dicephalosporamaetaengica* (MFLU 24-0330, holotype) **a** fresh apothecia growing on a dead wood **b**, **c** dried apothecia **d**, **e** vertical sections of the apothecia **f** excipulum **g** paraphyses and part of asci **h–j** asci (**i–j** asci in Meltzer’s reagent) **k–l** ascospores. Scale bars: 2 mm (**a–c**); 100 µm (**d, e**); 50 µm (**f–j**); 10 µm (**k, l**).

##### Material examined.

Thailand • Chiang Mai Province, MaeTaeng District, Pha Deng Village, Mushroom Research Center (MRC), altitude 863 m, on the decayed unidentified leaf, 18 August 2023, Le Luo, RB1 (MFLU 24-0330, holotype); • *ibid*., RB101 (MFLU 24-0331, paratype).

##### Notes.

*Dicephalosporamaetaengica* formed a clade closer to *D.menghaica* with 100% MLBS and 1.00 BIPP support (Fig. 1). These two species grouped as a sister clade and together formed a closely related clade to *D.albolutea* with 98% MLBS and 1.00 BIPP (Fig. 1) bootstrap support. *Dicephalosporamaetaengica* differs from *D.menghaica* by having larger (1.5–3.3 mm vs. 1–1.6 mm), yellow apothecia, long stipe (1.2–2.5 mm vs. 0.6–1 mm), slightly hyaline to light yellow at the base of stipe, 0–1–septate ascospores with paraphyses that are unbranched and aseptate, while *D.menghaica* has smaller apothecia, shorter stipe and slightly hyaline to light yellow at the base with branched paraphyses. *Dicephalosporaalbolutea* differs from *D.maetaengica* in having yellowish-white apothecia, longer asci (140–156 × 9.5–10.5 µm vs. 81–111 × 5.6–10.7 µm) and ascospores (26–31 × 3.8–5 µm vs. 14.5–21.8 × 2.4–4.2 µm) with aseptate paraphyses and ascospores (Zheng and Zhuang 2019). Therefore, *D.maetaengica* is introduced here as a new species.

#### ﻿ Dicephalospora menghaica

Taxon classificationFungiHelotialesHelotiaceae

L. Luo & K.D. Hyde sp. nov.

3B17D143-7221-5A4B-9B0C-1F98C2EE73FF

Index Fungorum: IF902891

Facesoffungi Number: FoF16775

Fig. 4

##### Etymology.

The epithet “*menghaica*” refers to the collection site, Menghai County, where the holotype specimen was collected.

##### Holotype.

HKAS135692.

##### Description.

***Saprobic*** on a dead leaf. **Sexual morph**: ***Apothecia*** 1–1.6 mm diam. when dry, arising solitary, uniseriate on petiole of leaves. Stipe 0.6–1 mm height and light brown to brown at the base. ***Receptacle*** orange and cupulate. ***Margins*** smooth and yellow when fresh. ***Disc*** slightly sunken and light yellow. ***Ectal excipulum*** 21–37 µm (x̄ = 28 µm, n = 60), multi-layered, thin-walled, with hyaline to light yellow cells of ***textura porrecta***. ***Medullary excipulum*** 24–50 µm (x̄ = 41 µm, n = 60), composed of thin-walled, hyaline to yellowish, gelatinized cells of ***textura intricata*** to ***globulosa***, small cells condensed. ***Hymenium*** 83–166 µm (x̄ = 122 µm, n = 60), hyaline to yellowish and inner mixed with asci and paraphyses. ***Paraphyses*** 0.8–2.1 µm wide (x̄ = 1.2 µm, n = 60), at the terminal cell, filiform, numerous, and have lengths exceeding the asci, smooth, branched, aseptate, slightly swollen, small, and globose at the apex. ***Asci*** (79–)82–125(130–) × 6.5–11.6 µm (x̄ = 101 × 9.4 µm, n = 50), 8-spored, unitunicate, cylindrical, and clavate, with the amyloid (J+) having a rounded apex, tapered, long stipitate base. ***Ascospores*** (16–)18–24(26–) × 2–3.7 µm (x̄ = 22 × 2.9 µm, n = 60), uniseriate to biseriate, fusoid-clavate with rounded ends, 7-septate, narrowed ends capped with pigments. **Asexual morph**: Not observed.

**Figure 4.**
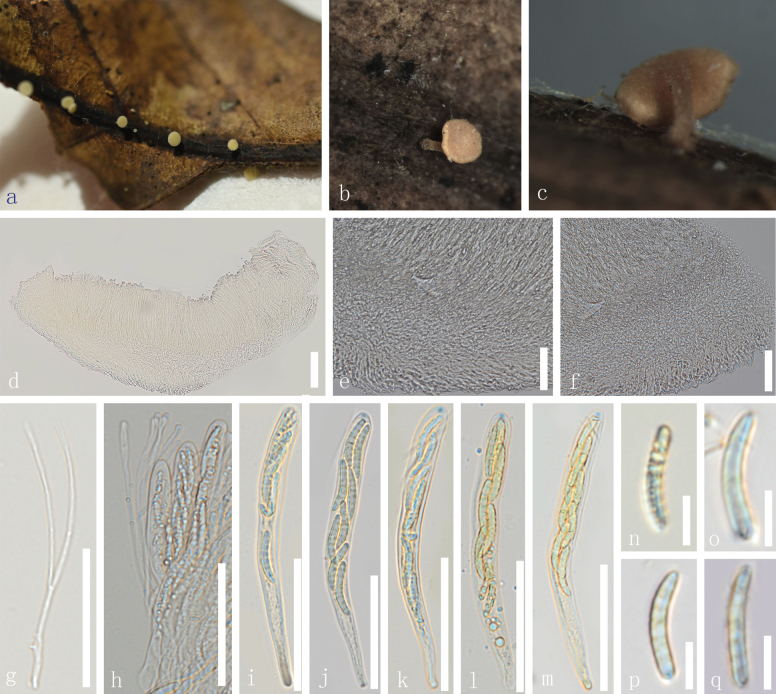
*Dicephalosporamenghaica* (HKAS 135692, holotype) **a** fresh apothecia growing on a dead wood **b, c** dried apothecia **d** a vertical section of the apothecium **e, f** excipulum **g** paraphyses **h** paraphyses and part of asci **i–m** asci (**j–m** asci in Meltzer’s reagent) **n**–**q** ascospores. Scale bars: 3 mm (**a**); 1 mm (**b, c**); 100 µm (**d**); 50 µm (**e–m**); 20 µm (**n–q**).

##### Material examined.

China • Yunnan Province, Xishuangbanna City, Menghai County, altitude 1595 m, on the decayed unidentified leaves, 8 September 2022, Le Luo, ly958 (HKAS 135692, holotype); • *ibid*., ly972 (HKAS 135690, paratype).

##### Notes.

*Dicephalosporamenghaica* clustered with *D.maetaengica* by 100% MLBS and 1.00 BIPP support (Fig. 1), and clustered sister to *D.albolutea* with 98% MLBS and 1.00 BIPP support (Fig. 1). *Dicephalosporamenghaica* differs from *D.albolutea* by having smaller (1–1.6 mm vs. 1–2.5 mm), light brown apothecia, slightly hyaline to light yellow at the base with branched, aseptate paraphyses, shorter asci (82–125 × 6.5–11.6 µm vs. 140–156 × 9.5–10.5 µm) and smaller ascospores (18–24 × 2–3.7 µm vs. 26–31 × 3.8–5 µm), whereas *D.albolutea* has cream to yellowish-white apothecia, with unbranched, septate paraphyses (Zheng and Zhuang 2019). *Dicephalosporamenghaica* also differs morphologically from *D.maetaengica* as discussed previously under the notes of *D.maetaengica*. Therefore, based on the morphological and phylogenetic analysis, *D.menghaica* is introduced here as a new species.

## ﻿Discussion

The members of Leotiomycetes are characterized by a diverse array of species with various ecological roles, including plant pathogens, endophytes, and saprobes (O’Brien et al. 2005; Sieber 2007; Baral 2016). They occur in aquatic to terrestrial ecosystems and play significant roles in decomposition and nutrient cycling (Quandt and Haelewaters 2021). In China, the diversity of Leotiomycetes is substantial due to the varied climates and ecosystems (Li et al. 2022; Su et al. 2023; Luo et al. 2024; Guo et al. 2024; Zhang et al. 2024). Ongoing research continues to uncover new species and understand their roles in ecosystem functioning, highlighting the importance of preserving fungal diversity for ecological health and agricultural sustainability (Li et al. 2022; Su et al. 2023; Luo et al. 2024).

The identification of *Dicephalospora* species has traditionally relied on morphological features, such as the color of the apothecia, anatomical structures, and the characteristics of asci and ascospores (Zheng and Zhuang 2019; Phutthacharoen et al. 2022; Luo et al. 2024). However, morphology-based identification became challenging due to the morphological variability observed even within the same species collected from the same geographical locations (Phutthacharoen et al. 2022). Morphological variation within a single fungal species can be influenced by genetic diversity, environmental conditions, transposable elements, and symbiotic relationships (Taylor et al. 2017; Senanayake et al. 2020). Similarly, apothecia and ascospores showed morphological diversity in *Dicephalosporarufocornea* isolates reported from the same province in Thailand (Phutthacharoen et al. 2022). This variability underscores the difficulty of relying solely on morphological characteristics for species identification and emphasizes the importance of integrating morphological data with molecular phylogenetics for accurate identification and classification. In this study, we updated the dichotomous key for the identification of *Dicephalospora* species.

DNA sequence data, particularly ITS and LSU gene sequences, play a crucial role in the delineation of fungal species (Hibbett et al. 2016; Jeewon and Hyde 2016). Our phylogenetic analyses based on these sequences provide robust support for the differentiation of the new species within *Dicephalospora*. The two new species, *D.menghaica* and *D.maetaengica*, are characterized by apothecial characters, with paraphyses that are either unbranched or branched. The phylogenetic relationships among *Dicephalospora* species in our study concurred with those reported by Phutthacharoen et al. (2022), who identified one main cluster and another subclade comprising *D.chrysotricha* isolates closer to the *Hymenoscyphus* clade. This discrepancy highlights the need for further research and more extensive sampling to clarify these relationships.

This study expands our understanding of *Dicephalospora*, particularly through the discovery and description of three new species from Xishuangbanna, Yunnan Province, China, and northern Thailand. *Dicephalospora* species are primarily saprobic, decomposing organic matter such as rotten wood, twigs, and leaf petioles. This saprobic activity plays a crucial role in nutrient cycling within their ecosystems. The tropical monsoon climate of Xishuangbanna, with its high humidity, provides a unique habitat for fungi, exemplified by the discovery of these species on wet decaying wood (Luo et al. 2024). This finding contrasts with previous observations that *Dicephalospora* were typically found in highly humid and cold areas, suggesting a broader ecological range and adaptability to various climatic conditions (Phutthacharoen et al. 2022). This study expands the *Dicephalospora* species up to 22 species, of which 16 were reported from China, four from Thailand (*D.chiangraiensis*, *D.irregularis*, *D.inthanonensis*, *D.maetaengica*), one from New Zealand (*D.chrysotricha*), and one from Australia (*D.sagerae*) (Spooner 1987; Zhuang et al. 2016; Zheng and Zhuang 2019; Phutthacharoen et al. 2022; Luo et al. 2024). China appears to harbor a high diversity of *Dicephalospora* species that are yet to be discovered.

This study contributes to the taxonomy and phylogeny of *Dicephalospora* by describing new species and clarifying their relationships within the Helotiaceae. In addition, a dichotomous key to the species in *Dicephalospora* is presented. Future studies should aim to include a wider range of genera and utilize additional genetic markers to further resolve the phylogenetic position of *Dicephalospora* and related taxa.

### ﻿A dichotomous key to the species of *Dicephalospora*

**Table:** 

1	Receptacle surface with hairs	***D.chrysotricha***
–	Receptacle surface without hairs	**2**
2	Sessile apothecia	**3**
–	Stipitate apothecia	**5**
3	Asci J^+^	***D.calochroa***
–	Asci J-	**4**
4	Disc concave with unbranched paraphyses	***D.sessilis***
–	Disc slightly convex with branched paraphyses	***D.irregularis***
5	Margin dentate	***D.dentata***
–	Margin not dentate	**6**
6	Disc cream to yellowish, white apothecia	***D.albolutea***
–	Disc and apothecia concolorous	**7**
7	Paraphyses with dark pigment contents	***D.phaeoparaphysis***
–	Paraphyses without dark pigment contents	**8**
8	Asci J-	**9**
–	Asci J^+^	**11**
9	Stipe base dark	***D.pinglongshanica***
–	Stipe base not dark	**10**
10	Apothecia orange when fresh, light brown when dry	***D.tengyueica***
–	Apothecia yellow to orange when fresh and dry	***D.xishuangbannaensis***
11	Ascospore cap mucilaginous	**12**
–	Ascospore cap non-mucilaginous	**13**
12	Ascospore lemon-shaped, 9−12.7 µm wide	***D.damingshanica***
–	Ascospore fusoid	***D.rufocornea***
13	Ascospores constricted in the middle	***D.contracta***
–	Ascospores not constricted in the middle	**14**
14	Ascospores elliptical-subfusoid	***D.shennongjiana***
–	Ascospores fusoid	**15**
15	Disc convex	***D.inthanonensis***
–	Disc flat	**16**
16	Paraphyses septate	**17**
–	Paraphyses aseptate	**18**
17	Ascospores multiseriate, ectal excipulum globose at the tips	***D.huangshanica***
–	Ascospores biseriate, 16.5−25.3 × 3.3−3.5 µm	***D.yunnanica***
18	Paraphyses branched	***D.menghaica***
–	Paraphyses unbranched	**19**
19	Ascospore aseptate	***D.aurantiaca***
–	Ascospore septate	**20**
20	Stipe and apothecia concolorous	***D.maetaengica***
–	Stipe and apothecia not concolorous	***D.chiangraiensis***

## Supplementary Material

XML Treatment for Dicephalospora tengyueica

XML Treatment for Dicephalospora maetaengica

XML Treatment for Dicephalospora menghaica

## ﻿Additional information

### Author ORCIDs

Le Luo https://orcid.org/0009-0006-7213-0498

Kandawatte Wedaralalage Thilini Chethana https://orcid.org/0000-0002-5816-9269

Qi Zhao https://orcid.org/0000-0001-8169-0573

Vinodhini Thiyagaraja https://orcid.org/0000-0002-8091-4579

Kitiphong Khongphinitbunjong https://orcid.org/0000-0002-4548-2790

Fatimah Al-Otibi https://orcid.org/0000-0003-3629-5755

Kevin D. Hyde https://orcid.org/0000-0002-2191-0762

### Data availability

All of the data that support the findings of this study are available in the main text.
